# Bicycling participation in people with a lower limb amputation: a scoping review

**DOI:** 10.1186/s12891-018-2313-2

**Published:** 2018-11-13

**Authors:** Jutamanee Poonsiri, Rienk Dekker, Pieter U. Dijkstra, Juha M. Hijmans, Jan H. B. Geertzen

**Affiliations:** 1 Department of Rehabilitation Medicine, University of Groningen, University Medical Center Groningen, CB41, PO Box 30001, 9700 RB Groningen, The Netherlands; 2 Department of Oral and Maxillofacial Surgery, University of Groningen, University Medical Center Groningen, Groningen, The Netherlands; 3 Sirindhorn School of Prosthetics and Orthotics, Faculty of Medicine Siriraj Hospital, Mahidol University, Bangkok, Thailand

**Keywords:** Bicycling, Lower limb, Amputation, Prosthesis, Motivation

## Abstract

**Background:**

To review literature on bicycling participation, as well as facilitators and barriers for bicycling in people with a lower limb amputation (LLA).

**Methods:**

Peer-reviewed, primary, full text, studies about bicycling in people with a LLA from midfoot level to hemipelvectomy were searched in Pubmed, Embase, Cinahl, Cochrane library, and Sportdiscus. No language or publication date restrictions were applied. Included full-text studies were assessed for methodological quality using the Effective Public Health Practice Project tool. Data were extracted, synthesized and reported following Preferred Reporting Items for Systematic Review.

**Results:**

In total, 3144 papers were identified and 14 studies were included. The methodological quality of 13 studies was weak and 1 was moderate. Bicycling participation ranged from 4 to 48%. A shorter time span after LLA and a distal amputation were associated with a higher bicycling participation rate particularly for transportation. In people with a transtibial amputation, a correct prosthetic foot or crank length can reduce pedalling asymmetry during high-intensity bicycling. People with limitations in knee range of motion or skin abrasion can use a hinged crank arm or a low profile prosthetic socket respectively.

**Conclusion:**

People with a LLA bicycled for transportation, recreation, sport and physical activity. Adaptation of prosthetic socket, pylon and foot as well as bicycle crank can affect pedalling work and force, range of motion, and aerodynamic drag. Because the suggestions from this review were drawn from evidences mostly associated to competition, prosthetists should carefully adapt the existing knowledge to clients who are recreational bicyclists.

**Electronic supplementary material:**

The online version of this article (10.1186/s12891-018-2313-2) contains supplementary material, which is available to authorized users.

## Background

In general bicycling has a number of physiological [1–4] and psychosocial benefits [5, 6]. Bicycling can, for instance, lower the risk of non-communicable diseases such as cardiovascular disease [1–3] and type 2 diabetes [7, 8]. Bicycling is thought to improve quality of life [9, 10]. People with a lower limb amputation (LLA) can also experience these benefits [9–11]. In addition, an increase in muscle strength of the intact and amputated limb as a result of regular bicycling [12] resulting in better walking [13, 14]. It is for the above mentioned reasons that enhancing the ability to perform physical activity (PA) such as bicycling for people with a LLA is important.

Bicycling is a low-impact activity as most of the body weight is supported by the bicycle’s seat, and consequently relieving the load on the residual limb in people with a LLA. But bicycling requires more degrees of flexion at the hip, knee and ankle than walking [15, 16] which could be limited by designs and functions of prosthetic components. Some reviews have been performed to gain information on barriers and facilitators in PA or sports participation in the group of physically disabled persons and people with a LLA, but not focusing on bicycling [17, 18]. One review provided a way to adapt prostheses and bicycles for bicyclists with a transtibial amputation (TTA), however, no information for other levels of LLA nor were participation rates reported [19]. Another review on bicycling for different amputation levels of lower and upper limbs included studies without people with a LLA and included studies with people cycling on ergometers [20]. These inclusions limit clinical relevance of outcomes of that review [20].

Assessment of bicycling participation, and associated facilitators and barriers can identify the needs of people with a LLA. That insight can assist clinicians and researchers to design interventions that meet with the clients’ goals and perspective and therefore may improve the participation in bicycling. Since bicycling has benefits, but information on participation in people with a LLA is lacking, the aim of this scoping review is to investigate and summarize bicycling participation rates in people with a LLA. The prevalence, frequency, duration and reasons for bicycling were identified. The second aim is to evaluate facilitators and barriers for bicycling in LLA.

## Methods

### Searches

Studies were searched in Pubmed, Embase, Cinahl, Cochrane library and Sportdiscus using a combination of Mesh terms and free texts. Part one of the search terms included MeSH terms and free texts relating to “amputee”, “amputation” and “prosthesis” and part two included terms related to “bicycling” or “sport”. Both parts were combined using “AND”. The search strategies were initiated by information specialist (librarian) with extensive expertise in systematic review searching. No time and language restrictions were applied. Last search date was March 22, 2018. This review follows Preferred Reporting items for Systematic Reviews and Meta-Analyses (PRISMA) [21]. In line with PRISMA full electronic search strategies of five databases was presented (Additional file 1).

### Participants

To be included, papers had to be about bicycling in people with a LLA either with or without a prosthesis, the minimum number of participants was one and the participants had to be human. At least one participant had to have a LLA from or proximal to midfoot level, but not above the hemipelvectomy level. Studies including multiple disabilities were only included when results for people with a LLA were reported separately. Papers were excluded if the participants use endoprostheses or implant devices.

### Types of studies to be included

All types of study designs which are a peer reviewed primary research and published as a full-text paper were included. Reviews, books, notes, letters to editors, expert opinions, conference abstracts or proceedings were excluded.

### Facilitators and barriers for bicycling

Factors influencing bicycling participation were classified into personal and environmental factors [22]. Any personal and environmental factors associated with bicycling were eligible. The personal and environmental factors associated with bicycling for all purposes were evaluated. The environmental factors make up physical, attitudinal and social environment in which people live such as prosthetic or assistive devices availability and access, infrastructure or policy [22]. The personal factors represent internal influences on functioning particular to the individual such as gender, motivation, self-efficacy, health status, or age [22]. Positive influences that help, motivate, or increase bicycling participation were considered facilitators. Negative influences that prevent, limit, or reduce bicycling were considered barriers.

### Primary outcome(s)

1. Bicycling participation (prevalence, frequency, and duration) which must be performed by a person with a LLA on a bicycle, not being an ergometer.

### Data extraction (selection and coding)

Two reviewers pilot tested assessments before each step of the review on papers not included in the review. Inter-rater agreement for titles and abstracts, and full text assessments were calculated using Cohen’s kappa (k).

$$ k=\frac{Po- Pe}{1- Pe} $$

P_0_ is the relative observed agreement between two reviewers, and P_e_ is the probability of chance agreement. K = 0 means there is no agreement, while K = 1 represents complete agreement between two reviewers. Low Cohen’s kappa (k ≤0.40) represents poor agreement between reviewers. Reviewers, in this case, may interpret and understand selection criteria differently. Two reviewers (JP& JHBG) assessed the titles and abstracts independently. Papers were selected for full text assessment if there was a part of the title or abstract referring to people with a LLA and bicycling or PA, sport, exercise, or training. Papers were excluded if titles or abstracts mentioned a specified PA that was not bicycling such as running, jogging, or walking. Only papers that were excluded by both reviewers did not proceed to the full-text assessment (reviewers were JP& JMH). The reference lists of included studies and of relevant reviews were assessed similarly on title and abstract and full text. Disagreement between reviewers in the full-text assessment was discussed until consensus was reached. If no consensus could be reached, a third reviewer gave a binding verdict (RD). Data was extracted by 2 reviewers (JP&PD) using a data extraction form developed for this study (Additional file 2).

### Risk of bias (quality) assessment

The quality of included studies was evaluated using the EPHPP (Effective Public Health Practice Project) tool. EPHPP tool was chosen due to the ability to assess the methodological quality of a range of study types regarding content validity and reliability [23–25]. Two reviewers (JP&RD) pilot tested the tool with excluded studies before assessing included studies. All studies were rated as strong, moderate or weak based on the rating of selection bias, study design, confounders, blinding, data collection method, and withdrawals and dropouts.

### Strategy for data synthesis

Characteristics of included studies (study design, year of publication, study country), participant characteristics, amputation level, cause of amputation, outcome measure, findings related to factors associated with bicycling, percentage of participants riding the bicycle, and bicycling frequency and duration were reported according to PRISMA [21] and presented in the summary of findings table. Meta-analysis was not performed since the included studies were heterogeneous with regard to study populations, intervention, measure, and outcomes.

## Results

### Selected studies

**Fig. 1:**
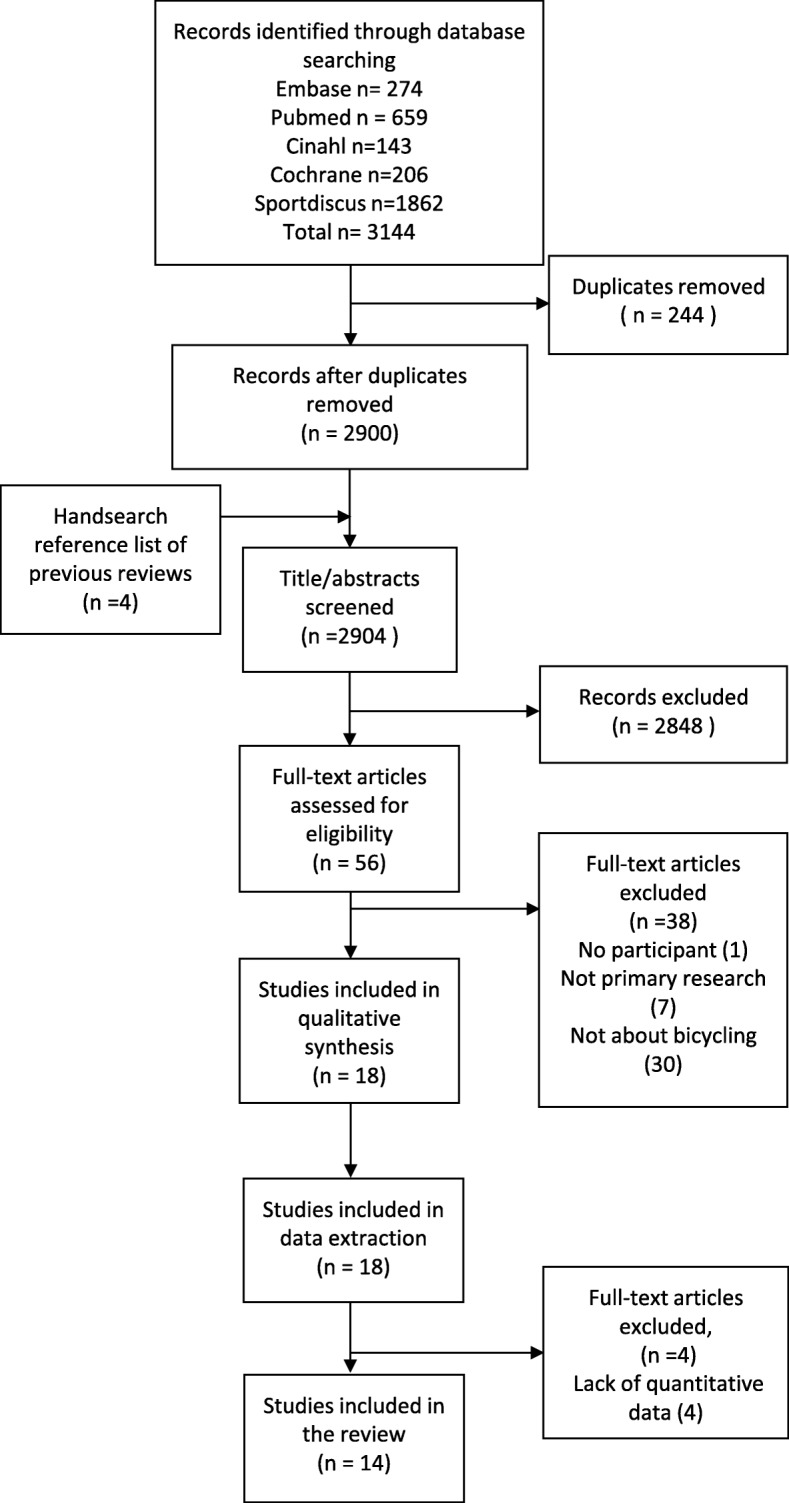
Flow diagram of studies inclusion and exclusion

After deduplication, 2904 titles and abstracts were screened of which 56 studies were included for the full-text screening (Cohen’s kappa = 0.761). Fourteen studies met the inclusion criteria [26–39] (Cohen’s kappa = 0.657) and were included for the quality assessment and data extraction (Fig. 1). One study was excluded because the study used mathematical model, so no participant in the study [40]. Seven articles were excluded because they were not primary research [41–47]. The other excluded studies were not about bicycling [48–75]. Full texts of 4 additional studies from the references of previous reviews and included studies were screened [26, 27, 76, 77]. Three of them passed to the full-text selection [26, 27, 76], two of which were about bicycling and therefore included for quality assessment and data extraction [26, 27].

Quantitative data relating to bicycling was not an inclusion criterion during titles and abstracts, and full text assessment. However, for data extraction, three studies were excluded due to the lack of quantitative data [78] and lack of separate reporting of information of people with a LLA [57, 79]. Sports popularity ranking was reported but not the number of people who took part in bicycling [78]. Participants of one study were grouped according to Para-cycling classification (C1-C5) which not only includes people with a LLA, but other types of impairments as well, such as hemiplegia, upper limb amputation, ataxia and spinal cord injury [79]. In addition, a study classified participants as physically challenged athletes including people with cerebral palsy, upper limb amputation, LLA and wheelchair users in which the people with a LLA could also be the wheelchair users [57]. Finally, a technical note about how to design a prosthetic shank was excluded due to a lack of quantitative data [80].

### The study design and quality

In total, 7 cross-sectional [27, 32–37], 4 case reports [26, 30, 38, 39], 2 cross-over trials [28, 29], and a cohort study [31] were included, investigating 1 to 780 of people with a LLA (Table 1). The majority of studies recruited participants from one source which was laboratory [26, 28, 29, 38, 39] or clinic/center [27, 32, 33, 37]. One study analyzed results of 2 Paralympic Games, and 5 World Championships [31]. Eleven studies had weak study design [26, 27, 30, 32–39]. Moderate and strong design were given to a cohort [31] and 2 cross-over trials respectively [28, 29]. Twelve studies did not report how possible confounders were controlled for and did not report reliability and validity of outcome measures [26–29, 32–39]. Six studies that reported the percentage of participants at data collection more than 80% were rated strong regarding the EPHPP for drop-outs. Following the guidelines of the EPHPP [81], all studies except one had an overall rating of weak.

**Table 1:** Quality of the included studies, based on the Effective Public Health Practice Project Tool

Ref	Selection Bias	Study Design	Confounders	Blinding	Data Collection	Drop-Outs	Global Rating
Narang et al. (1984) [27]	0	–	–	0	–	+	–
Burger et al. (1997) [32]	0	–	–	0	–	0	–
Burger et al. (1997) [33]	–	–	–	0	–	–	–
Mead(2005) [38]	–	-	–	0	–	+	–
Kars et al. (2009) [36]	–	–	–	0	–	–	–
Childers et al. (2011) [28]	–	+	–	0	–	+	–
Childers et al. (2011) [29]	–	+	–	0	–	+	–
Sprunger et al. (2012) [37]	–	–	–	0	–	–	–
Bragaru et al. (2013) [35]	–	–	–	0	–	–	–
Koutny et al. (2013) [26]	–	–	–	0	–	+	–
Littman et al. (2014) [34]	+	–	–	0	–	–	–
Scheepers (2015) [39]	–	–	–	0	–	+	–
Dyer and Woolley (2017) [30]	–	–	+	–	+	+	–
Dyer (2017) [31]	+	0	+	0	+	–	0
Totals							
weak(%)	10(71.4%)	11(79%)	12(86%)	12(86%)	12(86%)	6(43%)	13(93%)
moderate(%)	2(14.3%)	1(7%)	0	0	0	1(7%)	1(7%)
strong(%)	2(14.3%)	2(14%)	2(14%)	2(14%)	2(14%)	7(50%)	0

“Ref”: reference, “+”: strong, “0”: moderate, “-”: weak. The total at the bottom of Table 1 represents how weak, moderate and strong each criterion is

### **Bicycling participation** - prevalence, frequency, duration

**Fig. 2:**
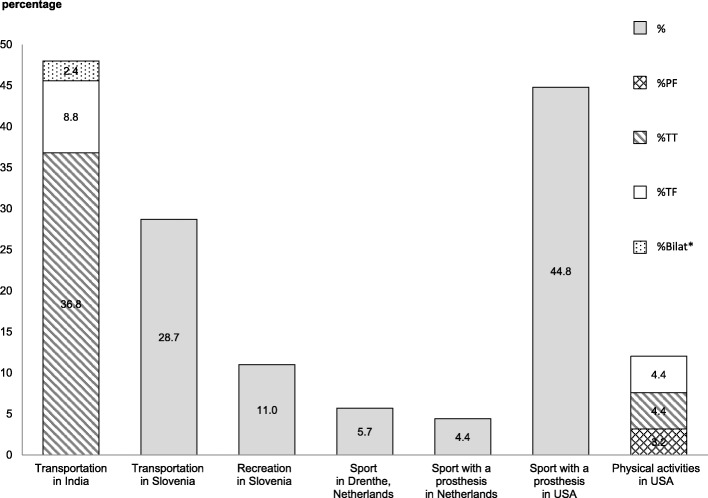
Percentages of people with a LLA bicycling for recreation and transportation in four countries. *PF* partial foot, *TT* transtibial, *TF* transfemoral, *Bilat* bilateral. *The level of amputation in the bilateral group was not reported in the study

Information about bicycling participation and purposes were extracted from 7 surveys published between 1984 and 2014 and included 58–780 participants. The participants varied in age and were mostly male (62–98%) (Table 2). For transportation, 29 and 48% of people with a LLA bicycled in Slovenia and India, respectively [27, 33]. In the United States 12–48%% of people with a LLA bicycled for recreation or PA, in Slovenia this was 11%, and in the Netherlands 4–6% for sport [32, 34–37](Fig. 2 and Table 2). No reports of frequency and duration found from the included studies.

**Table 2:** Data summary of included studies

Authors (year)	Country	Study design	LLA No, Male	Age (mean ± SD/ range)	Amp Characteristics			Results
					Cause	Level	Uni/Bilat	
Bicycling participation								
**Burger et al. (1997) [32]	Slovenia	CS	228, 84%	53.3 ± 15.4	100%T	108TF, 114TT, 2KD, 4HD	NR	Recreation: • Before amputation: 38% bicycling • After amputation: 11% bicycling
Kars et al. (2009) [36]	Netherlands	CS	105, 66%	23–79	40% PVD, 31% T, 10% C, 19% other	27TF, 58TT, 1Hemipelvectomy, 5HD, 13KD, 1 AD	101/4	Sport: • 6% bicycling for sport • A minimal duration of half an hour of participation is required for sports
Sprunger et al. (2012) [37]	USA	CS	58 (100%VA)	48.3 ± 14.3	88% T, 12% PVD, DM, C, or infection	22 Gr1, 26 Gr2, 10 Gr3	48/10	Sport: • 45% bicycling (most popular)
Bragaru et al. (2013) [35]	Netherlands	CS	780, 62%	59.6 ± 14.8	27% PVD/DM, 73% non-PVD	261TF, 432TT, 87KD	736/44	Sports with a prosthesis: • Athletes are persons who joined sport at least 5 h a month • 4% of participants were athletes who cycle with a prosthesis
Littman et al. (2014) [34]	USA	CS	158, 98%, (100%VA)	65	36% T, 64%-NR	41TF, 62TT, 55PF	125/33	Physical activities: • 12% bicycling outdoors or on stationary bicycle (9%of PF, 12%of TT and 17% of TF)
Bicycling participation and facilitators and barriers for transportation								
Narang et al. (1984) [27]	India	CS	500, 95% (60% VA)	2–65#	82% T, 17% disease, 1% congenital	124TF, 308TT	432/68	• 48% used bicycle (60% of TT, 35%of TF and 18% of bilat) • 50% did not use bicycle (38%of TT, 63% of TF, 78% of bilat) • 2% never known how to cycle (2% of TT and TF and 4% of bilat)
**Burger et al. (1997) [33]	Slovenia	CS	223, 84%	54.4 ± 15.4	100% T	102TF, 115TT, 2KD, 4HD	203/20	• 29% used bicycle • 60%*** did not use bicycle (average 5.7 years older than those who use a bicycle) • 11% did not travel by bicycle both before and after amputation • TT amputees were more likely to bicycle than TF amputees
Bicycling facilitators and barriers in people with a TTA								
Childers et al. (2011) [28]*	USA	RCT	8, 75% (1Paralympic medalist) (control =9)	36.4 ± 10.4	7 T, 1 C	8 TT	8/0	Pedaling force effectiveness ratio was not significantly different between a STIFF foot and a FLEX foot
Childers et al. (2011) [29]*	USA	RCT	8, 75% (1Paralympic medalist) (control =9)	36.4 ± 10.4	NR	8 TT	8/0	Pedaling asymmetry in people with a TTA was significantly larger than in controls in low difficulty and time trial conditions (submaximal bicycling over a 6-min period). Work asymmetry was significantly greater than the force asymmetry in TT amputation group between both conditions. Work and force was provided more by the sound limb. Work asymmetry decreased when the STIFF foot was used during the time trial condition.
Koutny et al. (2013) [26]	Czech Republic	CR	1, 100% (athlete)	37	NR	1TT	1/0	After shortening of the bicycle’s crank at the prosthetic limb, asymmetry of hip and knee kinematic reduced. Besides, muscle activity decreased during bicycling in seated position (vastus medialis, vastus lateralis, and gluteus maximus of both limbs) and climbing position (gluteus maximus of amputated limb). The sound side significantly produced more pedaling forces than the prosthetic side but this asymmetry was not influenced by the crank shortening.
Dyer and Woolley (2017) [30]	UK	CR	1, 100%	33	NR	1TT	1/0	An aero foil shaped pylon caused less, but not significant, aerodynamic drag than the round shaped pylon in both virtual elevation field and wind tunnel tests.
Dyer (2017) [31]	UK	Cohort	41,100%	NR	NR	41TT	41/0	The competitive bicyclists in C4 classification who used prosthesis were not faster when competing in 1 km time trial (world championships and Paralympic games) than the bicyclists without prosthesis.
Bicycling facilitators and barriers in Van Nes rotationplasty								
Mead (2005) [38]	Canada	CR	1, 100%	14	1 C	1 Van Nes rotationplasty	1/0	Limitation of knee flexion obstructed complete bicycling revolutions. By cutting a crank and adding a hinge in between two crank parts, the outer crank can swing down. The hinged-crank reduced amount of required knee flexion.
Scheepers et al. (2013) [39]	Netherlands	CR	1, 100%	18	1 C	1 Van Nes rotationplasty	1/0	The thigh cuff of a conventional prosthesis leads to perspiration, chaffing and skin abrasion in high-intensity bicycling. Replacing the thigh-cuff socket design and conventional prosthesis with the Socket-Less Rotationplasty Prosthesis for Cycling prevented abrasion.

“–” = weak, “0” = moderate; LLA = lower limb amputation; NO = number; SD = standard deviation; Amp = amputation; M = male; VA = veterans; Uni = unilateral; Bi = bilateral; CS = cross sectional; CR = case report; RCT = randomized control trial; PF = partial foot; TT = transtibial; TF = transfemoral; KD = knee disarticulation; HD = hip disarticulation; PVD = peripheral vascular disease; DM = diabetes; T = Trauma; C = Cancer; NR = not reported; Gr = group; Gr1 = TT and below; Gr2 = TF level and KD; Gr3 = above TF and all bilat; *’*,**,**have possibility of using the same group of participants in the studies of the same authors (Childers et al.*’* and Burger et al. **’**); *** the percentage reported from this review (60%) is different from the original study (62%); #Age of participants at the time of a LLA-57% of participants aged between 21 and 30 years old at the time of survey

### Bicycling facilitators and barriers

Table 2 presents factors associating to bicycling, bicycling purposes, and levels of amputation.

#### Transportation

Age, time since the LLA, and level of LLA were associated to bicycling participation [33]. People with LLA who bicycled were younger than who stopped bicycling [33]. People with longer period after LLA stopped bicycling more than the people with shorter period after LLA [33]. People with a TTA traveled by bicycling more than people with a transfemoral amputation (TFA) [27, 33]. Half of Indian participants stopped traveling by bicycle after LLA while a few percent of participants never knew how to cycle [27].

#### PA/ recreation/ sport

Having LLA and LLA level influenced bicycling for recreation. After LLA, people changed their recreational activities. Bicycling was the most popular activity before LLA, but the tenth after LLA [32]. The number of people with a TTA who bicycled was equal to TFA [34].

#### Competition

Three studies reported bicycling biomechanics in people with a TTA and the influence of adaptation of the prosthesis or the bicycle [26, 28, 29]. Pedaling work and force produced by the prosthetic and sound side were not the same, where the sound side contributed significantly more force [26, 29] and more work [28, 29] than the amputated side [26, 28, 29]. Pedaling asymmetry was also presented in able-bodied people, but to a smaller extent than in the people with a LLA [29]. When two prosthetic feet were compared, the aluminum or STIFF foot reduced work asymmetry more during high-intensity bicycling than the flexible carbon fiber dynamic response or FLEX-foot [29]. In low intensity, the FLEX and STIFF feet did not differ significantly in work asymmetry [29]. Furthermore, the ratio of forces orthogonal to the crank and the resultant force applied to the pedal which is called force effectiveness ratio were compared between the groups of able-bodied and people with a TTA. Pedaling force effectiveness ratio was not affected by the TTA or the applied prosthetic feet since the participants were able to compensate and achieve the overall force effectiveness by using their sound side [28].

The length of a crank arm also influenced hip and knee kinematics [26]. Shortening the crank arm reduced asymmetry in hip and knee angles between both limbs, and moreover, reduced the higher muscles activity in the prosthetic side [26]. In individuals with limited knee range, a hinged crank arm enabled the person to bicycle using also the affected side. The crank arm was cut and reattached with the hinge at an appropriate level [38]. Design of socket was found to be associated with skin abrasion in high intensity bicycling. To prevent the abrasion, a conventional prosthesis socket made of a leather thigh cuff was replaced by a socket-less prosthesis [39]. The shape of the pylon was associated with aerodynamic drag, however, the measures performed during the tests did not show significant differences of the aero foil shaped pylon compared to the round shaped pylon [30, 31]. Analysis of data from C4 bicyclists competing in the Paralympic Games and World Championships revealed no advantages of use of prostheses, in relation to those participants who did not use prostheses [31].

## Discussion

Health benefits from bicycling are apparent and bicycling serves as an alternative to other modes of transportation and exercise for LLA. After all, unlike running, skiing or golfing, recreational bicycling requires very little modifications to the people with a LLA to participate. Bicycling should remain an integral component of rehabilitation and the return to recreation and vocation [82]. Bicycling participation ranged from 4 to 48%. Two studies done in the same country reported considerable differences within a country [34, 37]. People with a LLA bicycled for reasons of transportation, recreation, sport and physical activity [27, 32–37]. Data about frequency and duration of bicycling were not found from the included studies. Since most studies aimed to determine changes of lifestyle or activities after LLA, intensity, duration and frequency of PA were reported for the big picture of attended activities but not specifically for bicycling [34, 37].

In this study, we assumed that the studies are about bicycling outdoor if it was not specified in studies [32, 34–37]. There is one study that the term bicycling referred to both indoor and outdoor bicycling [34]. When participation rate was reported, two studies reported sport participation if a minimum duration of half an hour for each participation [36] or total of 5 hours participation a month [35] was met. Whereas, in 5 studies, duration was no concern as long as the people with a LLA reported riding the bicycle [27, 32–34, 37]. Consequently, the participation rates should be interpreted with caution. Definitions of bicycling participation should be reported in future studies.

The majority of participants in the included studies were males with a TTA or TFA. Males with a TTA were reported to participate in the bicycling the most. The overall gender distribution of people with a LLA is about 50% male and female [83]. Therefore, including considerably more than 50% of male participants or veterans in studies [34, 37] may not represent the general population of people with a LLA. Although male, distal level of amputation and amputation due to trauma may associate to higher level of bicycling participation, the results of this review may not reflect the interest and purpose of bicycling of general populations with a LLA.

Bicycling was chosen as a form of transportation in Slovenia besides using cars and public transportations and was related to the level of independence [33]. A study from India investigated the functional capabilities of people after LLA and only included essential activities for daily living, vocational activities, and living arrangements in which bicycling was surveyed. It was demonstrated that bicycling was an important mode of transportation for civilians in India [27]. Both studies were done some decades ago; however, the recent studies did not show a difference in favor of other transportation modes in India especially in the group of low-income people [84, 85]. Socioeconomic status such as income or occupation may influence traveling by bicycling. A small number of participants could not ride the bicycle even before the amputation [27]. Knowledge or skill may facilitate bicycling participation. Two studies showed older age and longer time span after amputation [33] and level of LLA [27, 33] related to the reduce or stop traveling by bicycling. A change in lifestyle after the LLA was also reported in which bicycling became less popular after the LLA [29]. Disability, health, lack of energy and fatigue were personal barriers which were reported in a previous review of sports barriers and facilitators in adults with different types of physical disabilities [17]. It is possible that older people or a more proximal level of LLA may have more disabilities, health problems, less energy or more likely to be fatigue during bicycling. In contrast, for recreational bicycling, an equal number of veterans with a TFA and TTA was reported [34]. The relationship between amputation level and cause, these barriers and bicycling participation should be further studied.

Factors influencing competitive or high intensity bicycling were done in specific groups of bicyclists- TTA or Van Nes rotationplasty. Most studies investigated a small group of participants, mainly male, and having different bicycling experience [26, 28–30, 38, 39]. Only one study utilizing data of more than 8 participants (*N* = 41); however, the same participants were analyzed more than once [31].

For bicyclists with a TTA, the focus of research was towards asymmetry in force and work between sound and amputated side and ways to reduce this asymmetry for better performance [26, 28, 29]. Clinically, a STIFF foot reduced the pedaling work asymmetry in high-intensity or competitive bicycling [29] but not in recreational bicycling. In low-difficulty bicycling, no significant difference were found between the STIFF and the Flex foot suggesting a walking prosthetic foot may be adequate for recreational bicycling [29]. A pylon with an aerofoil shape caused less aerodynamic drag than the round shape [30]. On the one hand, a prosthetic foot and pylon influenced high intensity bicycling. On the other hand, while comparing between prosthetic and non-prosthetic bicyclists, there were no significant benefits reported from the prosthesis to the athletes [31]. Besides the kinetic asymmetry, there were hip and knee kinematic asymmetries which were reduced by shortening of the crank arm length on the amputated side [26]. The shortened crank arm also reduced the muscles activity on the amputated side [26]. Although the shortened crank arm improved the asymmetry in the person with a TTA in a case report [26], the same shortening may not give the same effects to other people with a TTA.

For a person with Van Nes rotationplasty, the socket design of walking prosthesis was a bicycling barrier because it caused skin abrasion [39]. Adding a hinge to the crank arm enabled a person with Van Nes rotationplasty with limited knee range of motion to bicycle [38]. Adaptation of the socket and crank arm might also facilitate bicycling in people with other types of LLA who have skin abrasions or limited knee ranges. The biomechanical effects from the prosthetic feet and crank arm shortening [26, 28, 29, 38] were also reported in a previous review of cycling with an amputation [20]. That review focused on upper and lower limb amputee biomechanics, physiology, and assistive technology development and extracted data from expert opinions, reviews, and primary researches from 2004 to 2014 [20]. Articles without any participants and studies using ergometers were included [20], while the current review included only primary research of at least one human with LLA riding a normal bicycle from all publication years. Therefore, the objectives, methods and results of the previous review differ from the current review.

The personal factors for transportation by bicycling were age, time since LLA, and level of amputation. The prosthetic socket design can either be an environment facilitator or barrier for high-intensity bicycling. The STIFF foot and crank arm shortening were environmental facilitators for high-intensity bicycling. For people with a knee flexion limitation, the limitation was a personal barrier and the hinged crank arm was an environment facilitator. Other bicycling environment factors such as weather, cycling paths, physical and emotional support, individual and societal attitudes toward people with a LLA, public services, systems or policies were not reported.

### Strengths and weaknesses

The search from 5 databases ensured that all medical and sports science articles could be included in this review. Eventually, only 14 studies were included showing this topic has not been explored very extensively.

Due to the small number of included studies with various focuses we were not fully able to answer the research questions of our study. Additionally the included studies had a high risk of bias. Though we focused more on the regular LLA, there were two case reports of Van Nes rotationplasty included in the review. It is understood that this special technique of LLA is very rare and the study designs were case reports, so the findings should be considered as low level evidence. Moreover, in the biomechanical studies in people with a TTA, the number of participants was also low and likely to be the same group of participants in the studies that have the same authors [28, 29]. Subsequently, the findings of this review may not reflect the general population of people with a LLA.

## Conclusion

Multiple purposes of use of bicycles are exhibited in this study. After LLA, people stopped bicycle or changed leisure activity. Age, level of LLA, and time since LLA influenced bicycling participation. Although environmental factors were limited to prosthetic socket and pylon design, foot stiffness and bicycle’s crank arm, arisen predominantly from small groups of bicyclists with a TTA and Van Nes rotationplasty, and mainly benefit to competition, some practical advices can be given. For instance, prosthetists may try to shorten the crank or use stiff foot if pedaling asymmetry is present. To supply prosthetists with sufficient additional knowledge in compiling an optimal prosthesis for bicycling, more research is necessary. Efforts towards studying facilitators and barriers for bicycling in a population with a LLA who are not athletes can benefit the general population with a LLA [9–14]. Further investigation of prosthetic and bicycle components in either competitive or recreational bicycling should include more participants, thus providing strong evidence for implementations by prosthetists or sport scientists.

## Additional files

Additional file 1: Search terms and number of articles found from five databases (last search done in Mar 22, 2018). (DOCX 39 kb)

Additional file 2: Data Extraction Form for Experimental /Observational Studies. (DOCX 18 kb)

## Abbreviations

EPHPP: Effective Public Health Practice Project
LLA: Lower limb amputation
PA: Physical activity
PRISMA: Preferred Reporting Items for Systematic Reviews and Meta-Analyses
TFA: Transfemoral amputation
TTA: Transtibial amputation
