# Supplementary material for: Bicycling participation in people with a lower limb amputation: a scoping review
Source: BMC Musculoskelet Disord. 2018 Nov 13;19:398. doi: 10.1186/s12891-018-2313-2 (PMC6234608; doi:10.1186/s12891-018-2313-2)
Supplement: Supplementary file 1 — Search terms and number of articles found from five databases (last search done in Mar 22, 2018). (DOCX 39 kb) [file 12891_2018_2313_MOESM1_ESM.docx]

**Additional file 1:**

Search terms and number of articles found from five databases (last search done in Mar 22, 2018)

| **Data base** | **Search** | |
| --- | --- | --- |
| Embase | 'amputee'/exp OR 'limb prosthesis'/exp OR amput*:ab,ti OR prosthet*:ab,ti OR prosthes*:ab,ti AND 'cycling'/exp OR bicycling:ab,ti OR cycling:ab,ti OR bike*:ab,ti OR cyclist*:ab,ti OR sport*:ab,ti AND [embase]/lim NOT [medline]/lim | |
| Pubmed | (("Amputees"[Mesh] OR amput*[tiab] OR "Artificial Limbs"[Mesh] OR prosthes*[tiab] OR prosthet*[tiab]) AND ("Bicycling"[Mesh] OR bicycling[tiab] OR cycling[tiab] OR bike*[tiab] OR cyclist*[tiab] OR sport*[tiab])) | |
| Cochrane library | #1  #2  #3  #4  #5  #6  #7  #8 | MeSH descriptor: [Bicycling] explode all trees  Bicycling or cycling or bike* or cyclist* or sport*:ti,ab,kw (Word variations have been searched)  amput* or Artificial Limb* or prosthes* or prosthet*:ti,ab,kw (Word variations have been searched)  MeSH descriptor: [Artificial Limbs] explode all trees  MeSH descriptor: [Amputees] explode all trees  #3 or #4 or #5  #1 or #2  #6 and #7 |
| Cinahl | S8 | S6 AND S7 |
|  | S7 | S1 OR S2 |
|  | S6 | S3 OR S4 OR S5 |
|  | S5 | TI (amput* OR prosthes* OR prosthet*) OR AB (amput* OR prosthes* OR prosthet*) |
|  | S4 | (MH "Limb Prosthesis") |
|  | S3 | (MH "Amputees") |
|  | S2 | TI (bicycling OR cycling OR bike* OR cyclist*) OR AB (bicycling OR cycling OR bike* OR cyclist* OR sport*) |
|  | S1 | (MH "Cycling") |
| Sportdiscus | S13 | S12 AND S9 |
|  | S12 | S1 OR S11 |
|  | S11 | AB bicycling OR cycling OR bike* OR cyclist* OR sport* |
|  | S10 | S3 AND S9 |
|  | S9 | S4 OR S5 OR S6 OR S7 OR S8 |
|  | S8 | SU artificial limbs |
|  | S7 | AB (amput* OR prosthes* OR prosthet*) |
|  | S6 | TI (amput* OR prosthes* OR prosthet*) |
|  | S5 | SU prosthesis or prosthetics |
|  | S4 | SU Amputees |
|  | S3 | S1 OR S2 |
|  | S2 | AB bicycling OR cycling OR bike* OR cyclist* |
|  | S1 | TI bicycling OR cycling OR bike* OR cyclist* |
