# Supplementary material for: Bicycling participation in people with a lower limb amputation: a scoping review
Source: BMC Musculoskelet Disord. 2018 Nov 13;19:398. doi: 10.1186/s12891-018-2313-2 (PMC6234608; doi:10.1186/s12891-018-2313-2)
Supplement: Supplementary file 2 — Data Extraction Form for Experimental /Observational Studies. (DOCX 18 kb) [file 12891_2018_2313_MOESM2_ESM.docx]

**Additional file 2:**

**Data Extraction Form for Experimental /Observational Studies**

**Reviewer** [ ] PD [ ] JP **Date of Review** _________________________________

**Author** ____________________________________ **Year** ____________________________

**Journal** __________________________________ **Ref ID** ____________________________

**Title**___________________________________________________________________________________________________________________________________________________

| **Study Method**   - RCT - Controlled clinical trial - Cohort analytic(two group pre+post) - Case-Control | - Cohort analytic(one group before and after) - Interrupted time series - Cross sectional - Case report |
| --- | --- |
| - others specify : ________________________________________ | |

**Study aim:** ___________________________________________________________________________

**Populations:** ___________________________________________________________________________

___________________________________________________________________________

­­­­**Participants** Setting: [ ] Hospital [ ] Clinic [ ] Community [ ] Others (………………………………..)

|  | **Race** | **%**  **male** | **%**  **female** | **Age**  **(range)** | **Cause of**  **amputation** | **amputated side** | | **Note:** |
| --- | --- | --- | --- | --- | --- | --- | --- | --- |
|  |  |  |  |  |  | Uni | Bi |  |
| **TF** |  |  |  |  |  |  |  |  |
| **TT** |  |  |  |  |  |  |  |  |
| **Others:** |  |  |  |  |  |  |  |  |

| **Intervention:** | **Sampler size :** |
| --- | --- |
|  |  |

Type of outcome

1. *Bicycling facilitators or barriers*
2. *Bicycling participation rate*
3. *Types of prosthetic, shoes , bicycles/components*
4. *Reasons of bicycling*

| **Outcome description** | **Scale/ measure** |
| --- | --- |
|  |  |

**Study results:**

**(a) Dichotomous data: page_______________**

| **Outcome** | **Intervention(__________ )**  **Number/ total number** | **Intervention(_________ )**  **number/total number** |
| --- | --- | --- |
|  |  |  |

**(b) Continuous data: page_______________**

| **Outcome** | **Intervention (____________ )**  **mean & SD (number)** | **Intervention (________ )**  **mean & SD (number)** |
| --- | --- | --- |
|  |  |  |

**Authors’ conclusion**__________________________________________________________ ____________________________________________________________________________________________________________________________________________________________________________________________________________________________________________________________________________________________________________

**Comments**___________________________________________________________________________________________________________________________________________________________________________________________________________________________________________________________________________________________________
